# THE EFFECT OF STRUCTURAL AND FUNCTIONAL SOCIAL SUPPORT OF OLDER KOREAN VOLUNTEERS ON LIFE SATISFACTION

**DOI:** 10.1093/geroni/igac059.2467

**Published:** 2022-12-20

**Authors:** Meeryoung Kim

**Affiliations:** Daegu University, Daegu, Kyongsang-bukto, Republic of Korea

## Abstract

As well as helping others, senior volunteers, like other seniors, need their own social support. However, unlike general older adults, older volunteers can build many social networks through volunteering. This study aims to examine how structural and functional social support of older volunteers affects their life satisfaction.This study used the sixth additional wave (2016) and the seventh wave (2017) of the Korean Retirement and Income Study. The subjects of this study were volunteers who are aged 60 and older, and the sample size was 241. Multiple regression was used for data analysis. Demographic variables were controlled. As for independent variables, structural and functional social support were used. For the dependent variable, life satisfaction was used. Structural social support was measured by the number of related subjects. For functional social support, Cohen and Syme (1985)'s social support scale, which includes instrumental, emotional, informational, and evaluative support, was used.According to the results of the study, structural support did not significantly affect the life satisfaction of older volunteers, but functional support had a significant effect on life satisfaction. Structural support was not significant because volunteers had many networks through volunteering. However, the functional social support of receiving practical help significantly increased the life satisfaction of the volunteers. These findings imply that having someone who can help them is an important factor in increasing life satisfaction.

